# Caffeine Enhances Memory Performance in Young Adults during Their Non-optimal Time of Day

**DOI:** 10.3389/fpsyg.2016.01764

**Published:** 2016-11-14

**Authors:** Stephanie M. Sherman, Timothy P. Buckley, Elsa Baena, Lee Ryan

**Affiliations:** ^1^Department of Psychology, Boston College, Chestnut HillMA, USA; ^2^Department of Psychology, University of Arizona, TucsonAZ, USA; ^3^Evelyn F. McKnight Brain Institute, University of Arizona, TucsonAZ, USA

**Keywords:** explicit memory, implicit memory, caffeine, time of day, cardiovascular exercise

## Abstract

Many college students struggle to perform well on exams in the early morning. Although students drink caffeinated beverages to feel more awake, it is unclear whether these actually improve performance. After consuming coffee (caffeinated or decaffeinated), college-age adults completed implicit and explicit memory tasks in the early morning and late afternoon (Experiment 1). During the morning, participants ingesting caffeine demonstrated a striking improvement in explicit memory, but not implicit memory. Caffeine did not alter memory performance in the afternoon. In Experiment 2, participants engaged in cardiovascular exercise in order to examine whether increases in physiological arousal similarly improved memory. Despite clear increases in physiological arousal, exercise did not improve memory performance compared to a stretching control condition. These results suggest that caffeine has a specific benefit for memory during students’ non-optimal time of day – early morning. These findings have real-world implications for students taking morning exams.

## Introduction

As any college student will tell you, the worst time to take a class is first thing in the morning. Unfortunately many classes and entrance exams are only offered during the time when most students are at their physiological low point of the day, as measured by body temperature, skin conductance, and heart rate (Horne and Ostberg, 1976; Bailey and Heitkemper, 2001). Importantly, this circadian slump comes with a cognitive cost. The majority of college students perform worse in the early morning compared to the afternoon on a variety of cognitive tasks that measure attention (Knight and Mather, 2013), learning (Anderson et al., 1991; Hidalgo et al., 2004), memory (Petros et al., 1990; May et al., 1993), and metamemory (Hourihan and Benjamin, 2013), skills that are critical for academic success. Many students rely on coffee – caffeine – to get them through those early morning exams. But does it actually help?

Caffeine is the most widely used stimulant, consumed daily by 80% of the world’s population and 90% of the North American population (Heckman et al., 2010). Caffeine is an efficient drug, crossing the blood-brain barrier quickly to block adenosine receptors that are distributed widely throughout cortical regions. Even at low doses, caffeine results in significant increases in firing rates in regions mediating sleep and mood, such as the dorsal and medial raphe nuclei and the locus coeruleus (Nehlig and Boyet, 2000). This amplified cortical activity likely underlies the increase in subjective reports of alertness (Smith, 2005; Michael et al., 2008) as well as increases in sustained attention and faster reaction times (Adan and Serra-Grabulosa, 2010; for review see: Einöther and Giesbrecht, 2012). These effects are amplified when individuals are experiencing low states of arousal as measured by subjective ratings of alertness (Brice and Smith, 2001) and sleepiness (Adan et al., 2008).

Surprisingly few studies, however, have shown that caffeine actually improves memory performance (Capek and Guenther, 2009; Borota et al., 2014; for review see: Nehlig, 2010), and even fewer studies have considered how caffeine interacts with time of day (Ryan et al., 2002; Walters and Lesk, 2015). In a previous study, we considered whether caffeine could overcome the well-documented decrease in memory performance among older adults during the late afternoon, when most older adults experience their circadian low point (for review see: May et al., 1993; Intons-Peterson et al., 1998; Hasher et al., 2005). We found that a single cup of coffee, ingested 30 min prior to memory testing, completely reversed the memory decline experienced by older adults in the afternoon (Ryan et al., 2002). We suggested that caffeine might influence memory performance by boosting general levels of arousal during non-optimal times of day.

Our previous study suggested two things. First, caffeine should have the same memory-boosting effect for young adults in the early morning, when most young adults are at their physiological low point. Second, we hypothesized that the effect was not due to caffeine specifically but should occur regardless of the method used to increase physiological arousal, such as engaging in physical exercise. In the present study, we tested these hypotheses by comparing the effects of caffeinated and decaffeinated coffee on college students’ memory performance at two times of day – early morning and late afternoon. To determine the specificity of the caffeine effect, we also compared two types of exercise in the early morning – vigorous aerobic exercise versus gentle stretching – on memory performance.

A secondary goal of the study was to consider the effect of caffeine on two different forms of memory, the deliberate recall of information (explicit memory) and the unintentional recall of previously learned information (implicit memory). Previous research suggests that while explicit memory scores are lower in young adults in the morning compared to the afternoon, priming scores actually increase during this period of low arousal, suggesting that explicit and implicit memory may be moderated differently by physiological arousal (May et al., 2005; Rowe et al., 2006).

Taken together, based on previous work, we expected that a) caffeine would boost explicit memory performance in young adults during the early morning while impairing priming, b) caffeine would have no effect on either explicit memory or priming during the afternoon, and c) vigorous exercise, but not gentle stretching, would have the same impact on memory performance as caffeine during early morning hours.

## General Methods

### Participants

Undergraduates from the University of Arizona (ages 18-21) participated in the study. Participants were excluded with a history of substance abuse or neurological or psychiatric disorders that might interfere with normal cognitive function. Prior to the study, participants were asked to estimate the number of cups of coffee, tea, sodas, sports/energy drinks, and chocolate bars they consumed weekly. Only those who consumed at least a moderate amount of caffeine on a weekly basis were enrolled in the study. Participants were also asked four key questions from the Morningness-Eveningness Questionnaire (MEQ; Horne and Ostberg, 1976) to exclude young adults who preferred mornings. Only 10% of students were excluded because they were morning-type individuals, consistent with prior studies using the MEQ (Horne and Ostberg, 1976; Chelminski et al., 1997). Participant characteristics for all three experiments are presented in **Table 1**. All participants provided written informed consent that was approved by the Institutional Review Board at the University of Arizona. In each experiment, participants were well matched on age, MEQ scores, and reported caffeine use (all *p*s > 0.05). Not surprisingly, number of hours slept the night prior to testing differed depending on the time of day. In Experiment 1, participants in the morning condition slept fewer hours compared to participants in the afternoon condition. This was demonstrated by a 2 × 2 ANOVA examining coffee type (caffeinated, decaffeinated) by testing time (morning, afternoon), indicating a main effect of time of testing, *F*(1,97) = 43.82, *p* < 0.01, but no effect of coffee type [*F*(1,97) = 0.97, *p* = 0.33]. In Experiment 2, the number of hours slept the night before testing between the exercise and stretching conditions did not differ, or between participants in Experiment 2 and the morning participants in Experiment 1 (*F*’s < 1).

**Table 1:** Participant characteristics for Experiments 1 and 2.

	Experiment 1				Experiment 2	
	Caffeine at non-optimal		Caffeine at optimal		Exercise at non-optimal	
Participant Data	time of day (morning)		time of day (afternoon)		time of day (morning)	
Condition	Caffeinated	Decaffeinated	Caffeinated	Decaffeinated	Exercise	Stretching
Participants	30	30	20	20	20	20
Age (years)	18.70 (0.92)	18.40 (0.77)	19.10 (1.25)	19.00 (1.38)	18.45 (0.92)	18.45 (1.0)
Sex (M/F)	10/20	15/15	8/12	7/13	3/17	5/15
MEQ scores	43 (6.65)	41(6.77)	44 (6.75)	44 (9.53)	43(4.76)	43 (7.12)
Hours of sleep night before	5.87 (1.48)	5.52 (1.24)	7.10 (1.36)	8.36 (1.80)	5.41 (1.22)	5.25 (1.40)
Daily caffeine intake (mg)	68.93 (48.62)	107.12 (119.10)	70.32 (56.12)	73.50 (42.79)	-	-

*Age, MEQ score, hours of sleep, and daily caffeine intake are expressed as the mean (SD). MEQ refers to the Morningness-Eveningness Questionnaire (Horne and Ostberg, 1976).*

### Materials

Seventy-two unique three-letter word-stems and completions (e.g., CAL____; CALCIUM) were chosen from a large normative word-stem completion dataset (Ryan et al., 2001). Each stem could be completed with a minimum of five words. Each completion was 5-9 letters in length and was not the most frequent completion for the stem. The lists were divided into three lists of 24 words, so that average word length and completion base rates were similar across the lists. Two lists were used during the study and test phase. The third list served as fillers during the tasks. The lists were counterbalanced across tasks and experimental conditions.

### Study Phase

Upon arrival, participants reported how awake they felt on a scale from 1 to 5 (1- not awake, 5- wide awake). After the experimental intervention (see specific experiments below), participants were shown 48 words (two of the three study lists) on a computer screen, one at a time. Two filler words were added at the beginning and end of the list to control for primacy and recency effects. Words were presented in random order for 3 s each. Participants were instructed to rate the pleasantness of the words on a scale from 1 to 5 (1- very unpleasant, 5- very pleasant) as the experimenter recorded their verbal responses. Participants were not informed that a memory test would follow later on.

During a 5-min interval, participants completed the full version of the MEQ and reported the time they went to bed the night before the experiment and when they woke up in the morning. They completed a second rating of how awake they felt and were asked whether they ate or drank anything before the experiment.

### Test Phase

In the test phase, the implicit memory task always preceded the explicit task. During the implicit word-stem completion task, thirty-six stems were presented on the computer screen, one at a time (e.g., BAS______). Twenty-four of the stems could be completed with words previously presented during the study phase, randomly intermixed with 12 word-stems from the non-studied list. Participants were instructed to complete the stem with the first word that came to mind. The explicit word-stem cued recall task followed immediately, consisting of the remaining 24 word-stems corresponding to words from the study list. Participants were instructed to complete the word-stems with words they saw earlier during the pleasantness-rating task. Priming was calculated by subtracting normative baseline completion rates collected by Ryan et al. (2001) from the percentage of stems completed with the words from the study phase. Cued recall was measured as the percentage of study words correctly recalled. After completing both memory tasks, participants provided a final rating of how awake they felt.

## Experiment 1 Morning Condition: The Impact of Caffeine at Non-Optimal Time of Day

### Participants and Procedures

Eligible participants were instructed not to eat or drink anything the morning of the experiment. They arrived at the laboratory between 6 and 7 a.m. and were randomly assigned to one of two conditions, caffeinated (*n* = 30) or decaffeinated (*n* = 30) coffee. Prepackaged instant coffee (Starbucks Via Italian Bold caffeinated and decaffeinated) was prepared using a standardized procedure to ensure that each 8-ounce cup contained the same amount of caffeine (approximately 180 mg in the caffeinated coffee, and 7-10 mg in the decaffeinated coffee; Starbucks Corporation).

Participants were given the cup of coffee to drink, and then read a book for 30 min. To control for expectancy effects, all participants were told that the coffee was caffeinated, and the experimenter was blind to the type of coffee administered. After 30 min, the implicit and explicit tasks were administered as described earlier. After completing the final wakefulness rating, participants were asked whether they felt that the coffee had affected them positively, negatively, or not at all.

### Results

Two participants, one from each group, were excluded because of extremely low explicit memory performance (only one and two correct answers), suggesting that they either did not understand the task or did not follow instructions. Another participant was dropped due to missing MEQ questionnaire data. To create an even number of participants in each condition, we randomly removed one additional participant in the decaffeinated group. The analyses were conducted on a final sample of 60 participants (30 in each group).

#### Wakefulness Ratings

**FIGURE 1:**
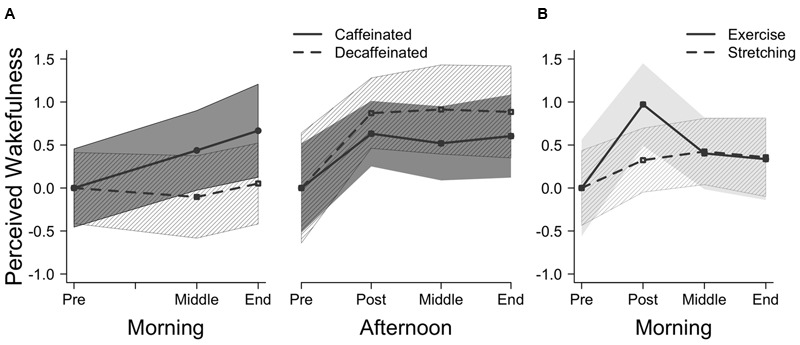
**Change in perceived wakefulness across different points in Experiment 1 (A)** and Experiment 2 **(B)**. Perceived wakefulness ratings are presented as *z*-scores to compare across experiments with 95% confidence bands. After the data were transformed to *z*-scores, each participant’s baseline perceived wakefulness rating was subtracted from their rating at each time point to illustrate their change in wakefulness across the experiment.

Participants who drank caffeinated coffee were significantly more awake by the end of the experiment, while participants who drank decaffeinated coffee did not experience the same increase in perceived wakefulness, as depicted in **Figure 1A** (Morning). This was demonstrated by a marginally significant interaction in a mixed-factor ANOVA between repeated wakefulness ratings (before coffee, intervening phase, and at the end of experiment) and between-group coffee type (caffeinated, decaffeinated), *F*(2,58) = 2.53, *p* = 0.08, $\eta_{p}^{2}$ = 0.04. Follow-up repeated measure ANOVAs indicated that wakefulness significantly varied across time in the caffeinated group [*F*(2,58) = 4.77, *p* = 0.01, $\eta_{p}^{2}$ = 0.14], but not in the decaffeinated group (*F* < 1, ns, $\eta_{p}^{2}$ = 0.01). Although baseline perceived wakefulness ratings did not differ between the groups *t*(58) = -1.52, ns, Cohen’s *d* = -0.39, CI [-0.90,0.12], paired *t*-tests indicated that the caffeinated group was significantly more awake at the end of the experiment compared to the beginning, *t*(29) = -2.52, *p* < 0.05, Cohen’s *d* = 0.46, CI [0.08,0.83]. Participants in the caffeinated group were also more likely than those in the decaffeinated group to report that the caffeine affected them positively (83% vs. 57%, respectively, *X*^2^(2) = 11.3, *p* < 0.01). No participant reported that caffeine affected him or her negatively.

#### Memory Performance

**FIGURE 2:**
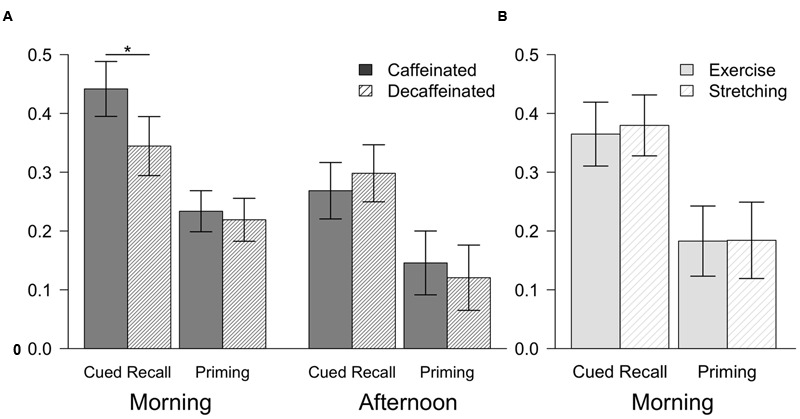
**Mean performance for cued recall and priming for Experiment 1 (A)** and Experiment 2 **(B)**. Cued recall was measured as the percentage of study words correctly recalled. Priming scores were calculated by subtracting normative baseline completion rates (Ryan et al., 2001) from the percentage of stems completed with the words from the study phase. Error bars show the 95% confidence intervals. ^∗^*p* < 0.05.

Participants in the caffeinated group performed significantly better than the decaffeinated group on word-stem cued recall, but the groups did not differ on priming. Results are depicted in **Figure 2A** (Morning). A mixed-factor ANOVA indicated a significant interaction between the repeated measure test type (explicit, implicit) and between-group coffee type (caffeinated, decaffeinated), *F*(1,58) = 4.53, *p* < 0.05, $\eta_{p}^{2}$ = 0.07. Follow-up *t*-tests illustrated that participants who ingested caffeinated coffee (*M* = 0.44, *SD* = 0.13) performed better than those who drank decaffeinated coffee (*M* = 0.34, *SD* = 0.13) on cued recall, *t*(58) = 2.90, *p* < 0.01, Cohen’s *d* = 0.75, CI [0.22,1.27]. However, implicit memory performance did not differ between the caffeine group (*M* = 0.23, *SD* = 0.09) and the decaffeinated group (*M* = 0.22, *SD* = 0.10), *t*(58) < 1, ns, Cohen’s *d* = 0.15, CI [-0.36,0.66].

Since the majority of the caffeine group (83%) but only a little over half of the decaffeinated group (57%) reported that caffeine had a positive effect, it is possible that the difference in explicit memory performance was driven by the participants’ perception of a benefit of caffeine, rather than the caffeine itself. We examined this possibility by comparing memory performance within the decaffeinated group between those who did and did not report a positive effect of coffee. Perception alone did not influence explicit performance; there was no difference between those who thought the caffeine affected them positively (*n* = 17, *M* = 0.31, *SD* = 0.10) and those who did not (*n* = 13, *M* = 0.39, *SD* = 0.15), *t*(28) = 1.58, ns, Cohen’s *d* = 0.58, CI [-0.16,1.32].

## Experiment 1 Afternoon Condition: The Impact of Caffeine at Optimal Time of Day

Consuming caffeine increased explicit memory performance for college-aged adults during early morning hours. Young adults who drank caffeinated coffee showed a 30% benefit in cued recall performance compared to the decaffeinated coffee drinkers, and this effect was independent of the perceived positive effect of the caffeine. The result is consistent with our previous study (Ryan et al., 2002) showing that older adults benefit from caffeine during their non-optimal time of day, which, for older adults, is the afternoon rather than early morning hours. However, caffeine had no effect on word-stem completion priming. The latter finding suggests that priming may not be influenced by manipulations of arousal.

Next, we considered whether caffeine would result in the same increase in explicit memory performance during the afternoon as observed in the morning. We expected that caffeine would not enhance explicit memory performance in the afternoon, since young adults are already at their physiological peak, and that caffeine would similarly have no effect on priming.

### Participants and Procedures

Forty-three undergraduates were randomly assigned to the caffeinated group or decaffeinated group. The procedures were identical to the morning session, except participants were tested between 2 and 4 p.m. Participants were instructed not to drink caffeinated beverages on the day of the experiment.

### Results

Three participants were excluded because of extremely low explicit memory performance (zero, one, and two correct answers), suggesting that they either did not understand the task or did not follow instructions. The analysis was conducted on a final sample of 40 participants (20 in each group).

#### Wakefulness Ratings

The caffeinated and decaffeinated groups did not differ in perceived wakefulness, indicated by a non-significant interaction between wakefulness ratings (pre-coffee, 30 min after the coffee, intervening phase, and at the end of the experiment) and coffee type (caffeinated, decaffeinated), *F* < 1, ns, $\eta_{p}^{2}$ = 0.02, and no main effect of coffee type, *F*(1,38) = 2.09, ns, $\eta_{p}^{2}$ = 0.05. Wakefulness ratings are depicted in **Figure 1A** (Afternoon).

Interestingly, participants who ingested caffeinated coffee were no more likely to report that the caffeine affected them positively (55%) than participants in the decaffeinated group (60%), *X*^2^(2) = 0.99, ns. Unlike the morning testing session, 25% of the caffeinated group reported that the coffee actually affected them negatively, while no one in the decaffeinated group reported a negative effect of coffee.

#### Memory Performance

In contrast to the morning testing session, caffeine did not influence either type of memory performance in young adults in the afternoon (**Figure 2A**) (Afternoon). Cued recall performance did not differ between those who ingested caffeinated coffee (*M* = 0.27, *SD* = 0.10) and decaffeinated coffee (*M* = 0.30, *SD* = 0.10), indicated by the lack of a main effect of coffee type, *F* < 1, ns, $\eta_{p}^{2}$ = 0.0002. Implicit memory performance also did not differ between the caffeinated (*M* = 0.14, *SD* = 0.12) and decaffeinated groups (*M* = 0.12, *SD* = 0.12), and there was no interaction between coffee type and memory test type (implicit, explicit), *F*(1,38) = 1.23, ns, $\eta_{p}^{2}$ = 0.03, suggesting that neither priming nor explicit memory were influenced by caffeine.

Again, we considered whether the perceptions of individuals regarding the positive effect of caffeine could have influenced the results, particularly since 25% of participants in the caffeine group reported a negative effect of caffeine. We compared those individuals reporting a positive or non-positive (neutral or negative) effect of caffeine separately for the caffeinated and decaffeinated groups. The decaffeinated group did not differ in explicit memory performance based on the perception of a positive effect (*n* = 12, *M* = 0.31, *SD* = 0.09) or non-positive effect (*n* = 8, *M* = 0.27, *SD* = 0.12) of caffeine, *t*(18) < 1, ns, Cohen’s *d* = 0.40, CI [-0.51,1.30]. The caffeinated group also did not differ in memory performance based their perceptions of the effects of coffee (positive: *n* = 11, *M* = 0.26, *SD* = 0.09; non-positive: *n* = 9, *M* = 0.28, *SD* = 0.12), *t*(18) < 1, ns, Cohen’s *d* = 0.20, CI [-0.69,1.08].

## Experiment 1: Directly Comparing Morning and Afternoon Testing Sessions

In order to better understand the impact of caffeine at different times of day, we compared participants in the morning and afternoon testing sessions on wakefulness measures and memory measures.

### Wakefulness Ratings

Ingesting caffeine in the morning differentially affected how awake participants felt by the end of the experiment compared to ingesting caffeine in the afternoon. A 2 × 2 ANOVA indicated an interaction between time of testing session (morning, afternoon) and coffee type (caffeinated, decaffeinated) on change in perceived wakefulness from the beginning to the end of the experiment, *F*(1,96) = 3.04, *p* = 0.08, $\eta_{p}^{2}$ = 0.03. Follow up *t*-tests suggested that while the morning (*M* = 0.67, *SD* = 1.45) and afternoon (*M* = 0.60, *SD* = 1.03) caffeinated groups did not differ in how awake they felt by the end of the experiment, *t* < 1, the decaffeinated group felt significantly more awake in the afternoon (*M* = 0.88, *SD* = 1.42) compared to the morning (*M* = 0.05, *SD* = 1.26), *t*(48) = -2.37, *p* < 0.05, Cohen’s *d* = 0.66, CI [0.10,1.26].

### Memory Performance

Data were first analyzed with a mixed-factor 2 × 2 × 2 ANOVA comparing test type (explicit, implicit), time of day (morning, afternoon), and coffee type (caffeinated, decaffeinated). Importantly, the 3-way interaction between test type, time of day, and coffee type was significant, *F*(1,96) = 4.86, *p* = 0.03. The omnibus test was followed up with separate ANOVAs on explicit and implicit tests.

Comparing the results of the explicit memory test across time of day, ingesting caffeine only improved explicit memory performance during the morning testing session. A 2 × 2 ANOVA indicated a significant interaction between time of testing session (morning, afternoon) and coffee type (caffeinated, decaffeinated) on explicit memory performance, *F*(1,96) = 6.71, *p* < 0.01, $\eta_{p}^{2}$ = 0.07. Although a main effect of testing time suggested that participants performed better in the morning overall [*F*(1,97) = 18.96, *p* < 0.01], this effect was driven by enhanced performance in the morning participants who ingested caffeinated coffee. Follow up t-tests showed that the decaffeinated groups did not differ in explicit memory between the morning and afternoon conditions, *t*(48) = 1.3, *p* = 0.20. These results further illustrate that the impact of caffeine on memory performance depended on the time of the testing session – explicit memory was only enhanced by caffeine during the morning testing session.

In contrast, caffeine did not differentially affect implicit memory across the morning and the afternoon testing sessions, indicated by a non-significant interaction between time of testing and coffee type, *F* < 1. Regardless of coffee type, participants had higher implicit memory performance (priming) in the morning compared to the afternoon. This was demonstrated by a main effect of time of day, *F*(1,97) = 19.17, *p* < 0.01, $\eta_{p}^{2}$ = 0.17.

## Experiment 2: Exercise at Non-Optimal Time of Day

As hypothesized, caffeine did not affect explicit memory during young adults’ optimal time of day, the afternoon, consistent with the notion that caffeine is only effective when physiological arousal levels are low. Additionally, caffeine had no effect on priming scores. In Experiment 2, we consider whether the observed improvement in explicit memory performance was due specifically to the ingestion of caffeine, or from the non-specific effect that caffeine has on increasing physiological arousal.

To determine the specificity of the caffeine effect, participants in Experiment 2 engaged in cardiovascular exercise during the early morning, their non-optimal time of the day. Even short sessions of exercise reliably elevate physiological arousal (Hung et al., 2013). Acute exercise increases the concentration of catecholamines in the brain including dopamine, epinephrine, and norepinephrine in the locus coeruleus (Cooper, 1973; McMorris et al., 2011). Just as caffeine acts on the locus coerulus to increase wakefulness, exercise increases norepinephrine in the locus coeruleus, which in turn induces arousal (Dietrich and Audiffren, 2011). If caffeine benefits explicit memory by increasing general physiological arousal, we would expect to see the same boost in memory performance after morning exercise. Since previous work suggested that implicit memory is optimal during low arousal times of the day (May et al., 2005), we expect exercise to increase arousal resulting in decreased priming.

### Participants and Procedures

Forty undergraduates participated in a session conducted between 6 and 8 a.m. Participants were randomly assigned to an exercise condition (*n* = 20) or a gentle stretching condition (*n* = 20). They were instructed not to eat or drink anything but water on the morning of the experiment. Participants were informed they could withdraw at any time if they were unable to complete the required exercise.

Upon arrival, participants provided a wakefulness rating on a scale from -5 (not awake) to 5 (wide awake). Then, they were equipped with an activity watch (New Balance Duo Sport Monitor, Durham, NC) that records heartbeats per minute. Participants practiced measuring their heart rate with the watch. A baseline heart rate measure was taken once the participant could operate the device.

Participants completed approximately 15 min of cardiovascular exercise (exercise group) or a gentle stretching routine (stretching group). For the exercise group, cardiovascular exercise was defined as 10 min of exercise with a 20 percent or greater increase in heart rate from baseline. Participants achieved this by performing interval laps of running up a set of stairs and briskly walking down another set of stairs at the other end of the hallway. An experimenter was stationed at the end of each lap to record heart rate. In the stretching group, participants completed 15 min of a simple stretching routine. Experimenters demonstrated all the stretches and recorded heart rates at 1-min intervals. After completing either the exercise or stretching protocol, participants gave a second rating of wakefulness and returned to the laboratory for memory testing.

### Results

#### Heart Rate

Heart rate was calculated as the average across 15 min of activity and compared to baseline heart rate. Participants in the exercise group experienced a substantial increase in heart rate from baseline compared to the stretching group, demonstrated by a significant group (exercise, stretching) by heart rate (baseline, activity) interaction, *F*(1,38) = 137.5, *p* < 0.0001, $\eta_{p}^{2}$ = 0.78. While baseline heart rate did not differ between the exercise (*M* = 80.55, *SD* = 14.09) and stretching groups (*M* = 80.65, *SD* = 13.20), *t*(38) < 1, Cohen’s *d* = 0.007, CI [-0.61,0.63], during activity the exercise group experienced significantly higher heart rates (*M* = 139.28, *SD* = 16.54) relative to the stretching group (*M* = 90.70, *SD* = 10.90), *t*(38) = 10.96, *p* < 0.00001, Cohen’s *d* = 3.47, CI [2.46,4.45].

#### Wakefulness Ratings

Participants who completed the exercise protocol reported feeling significantly more awake immediately following the activity compared to the stretching condition, depicted in **Figure 1B**. The perceived wakefulness (pre activity, post activity, intervening phase, and at the end of experiment) by group (exercise, stretching) interaction was significant *F*(1,38) = 2.73, *p* < 0.05, $\eta_{p}^{2}$ = 0.07, demonstrating that the perceived wakefulness of the participant depended on whether they completed 15 min of stretching or exercise. Although baseline perceived wakefulness ratings did not differ between the groups *t*(38) = 1.09, ns, Cohen’s *d* = 0.34, CI [-0.28,0.97], follow-up paired *t*-test indicated that the exercise group reported a significant increase in wakefulness immediately following exercise compared to their baseline wakefulness rating, *t*(19) = -4.25, *p* < 0.0001, Cohen’s *d* = 0.95, CI [0.41,1.47].

#### Memory Performance

Exercise did not have an impact on either explicit or implicit memory during the early morning, despite participants feeling more awake after exercise (**Figure 2B**). No interaction between group (exercise, stretching) and memory type (implicit, explicit) was observed, *F* < 1, ns, $\eta_{p}^{2}$ = 0.002. Explicit memory performance did not differ between those who completed the exercise (*M* = 0.36, *SD* = 0.18) and stretching protocols (*M* = 0.38, *SD* = 0.11), *t* < 1, ns, Cohen’s *d* = 0.13, CI [-0.49,0.75]. Similar results were seen for implicit memory across the exercise (*M* = 0.18, *SD* = 0.13) and stretching protocols (*M* = 0.18, *SD* = 0.14), *t* < 1, ns, Cohen’s *d* = 0.01, CI [-0.61,0.63].

## General Discussion

Consuming caffeinated coffee results in significantly higher memory performance on an explicit cued–recall task in the early morning, but not in the late afternoon. These results are consistent with the hypothesis that caffeine benefits cognition during suboptimal conditions (Ryan et al., 2002; Nehlig, 2010) - in this case, during the low point in young adults’ circadian rhythm. Importantly, the benefits of caffeine for explicit memory performance do not appear to be related to an acute increase in physiological arousal (Experiment 2), to the perception of being more awake and energized after ingesting coffee (Experiment 1), or general expectancy effects since participants in Experiment 1 were all told they were consuming caffeinated coffee.

We were somewhat surprised by the finding that elevating arousal through exercise during the morning did not improve explicit memory performance, particularly since participants consistently reported feeling more awake and energized after exercise. Our finding is consistent, however, with research suggesting that the cognitive benefits of exercise build gradually, rather than acutely. For example, Bugg et al. (2006) found that older adults who engage in an active lifestyle do not experience a decline in working memory performance across time-of-day compared to sedentary older adults. These authors argue that habitual exercise leads to increased calcium levels, which are necessary for the metabolism of dopamine and norepinephrine. This increase in calcium occurs gradually and is maintained through consistent exercise. In contrast, caffeine results in a fast blockade of adenosine receptors, preventing the blockade of norepinephrine (McGaugh, 2000), which may influence the consolidation of new memories. The difference in the timeline of the effects of caffeine and exercise may explain why a single cup of coffee benefits memory and acute exercise does not.

Given that participants were not morning-type individuals, it is not surprising that they tended to go to bed late even though they were scheduled for the early morning testing condition. Accordingly, the young adults tested during their non-optimal time of day (the morning) reported fewer hours of sleep than those who were tested during their optimal time of day in the afternoon. The difference in sleep time between the morning and afternoon testing conditions likely reflects the real-world situation for college students. This decrease in sleep adds to, or may even account for, the impact of circadian rhythms on cognitive functioning in young adults. Importantly, however, morning participants did not differ in the number of hours slept between the caffeinated and decaffeinated conditions, and these individuals did not differ from participants in the exercise and stretching conditions. The only difference between all morning testing groups that had an impact on memory performance was the administration of caffeine.

The mechanisms by which caffeine enhances explicit memory remain unclear. Recently, Borota et al. (2014) suggested that caffeine has a specific effect on memory consolidation. They found that caffeine ingested immediately *after* studying a series of object pictures resulted in better discrimination between old objects and visually similar lures, but not better recognition performance *per se*, suggesting a specific effect of caffeine on pattern separation. Consistent with our findings, the authors conclude that the benefit of caffeine is not merely due to general increases in arousal and attention. Memory enhancement may be mediated by increases in levels of norepinephrine that have been shown to benefit pattern separation (Segal et al., 2012) or by enhancing long-term potentiation in the hippocampus due to the high concentration of adenosine receptors in the CA2 region (Simons et al., 2012). This explanation, focusing on caffeine’s potential enhancement of hippocampally mediated memory, is consistent with our finding that caffeine benefits explicit, but not implicit, memory. Multiple forms of implicit learning, including repetition priming, are thought to rely on cortical adaptations that are independent of the hippocampus (for review, see Reber, 2013).

This cannot be the whole story, however. The striking differential effect of caffeine in the early morning versus late afternoon suggests that caffeine’s efficacy interacts with other factors. It could be that participants in afternoon caffeine experiment did not follow our instructions to refrain from consuming caffeinated beverages on the day of the experiment, and thus an additional 200 mg of caffeine made no difference to memory performance. Alternatively, other physiological factors that vary naturally with circadian rhythms may result in a system that is already “optimized”, so that caffeine either has no benefit, or may even result in decreased performance when taken in sufficiently large doses (Borota et al., 2014). A third possibility, discussed earlier, is that caffeine was effective because morning participants had fewer hours of sleep the night before testing, compared to afternoon participants. Adan et al. (2008) found that a low dose of caffeine decreased self-reported sleepiness in the morning, and sleepiness improved even more among evening-type participants, which would match the participants included in the present study. However, Adan et al. (2008) did not assess cognitive functioning, so whether sleepiness, independent of circadian rhythms, was sufficient to impact cognitive functioning is not known.

It is important to note, however, that unlike our previous work examining memory and time of day in older adults (Ryan et al., 2002), we did not find the expected time of day effect, which should have resulted in better cued recall performance in the afternoon compared to the morning. Instead, explicit memory performance did not differ between the decaffeinated groups in the morning and afternoon. Notably, we chose not to use a within-subjects design as we did in our previous study with older adults because the implicit memory task could only be performed once without the participants’ conscious awareness (Delpouve et al., 2014). It is unclear whether testing the same individuals in the morning and the afternoon would have shown the expected time of day effect in explicit memory, although we note that other researchers have reported the effect using between-subjects designs (e.g., May et al., 1993). In line with previous work, we demonstrated that implicit memory was higher during participants’ non-optimal time of day (May et al., 2005; Rowe et al., 2006; Delpouve et al., 2014), although priming was not influenced by caffeine either during the morning or the afternoon.

In summary, our results suggest that caffeine results in explicit memory enhancement for young adults during their non-optimal time of day – early morning. Although it is well documented that very few young adults perform best in the morning (Chelminski et al., 1997), many standardized tests and final exams are taken within the first few hours of the school day. Most college instructors simply assume that grades on these tests accurately reflect a student’s ability, but this is likely not the case. Several studies in academic settings suggest that a student’s time of day preference impacts overall academic performance. For example, Randler and Schaal (2010) found that grade point average was negatively correlated with MEQ scores – the more a student preferred evening hours, the worse grades they earned in school. The degree to which this effect is due specifically to differences in circadian rhythms or the lack of sleep that likely occurs among these students is unclear. Nevertheless, it appears that for these students, caffeine has a benefit for learning. It remains to be seen whether consuming caffeine would result in better learning, whether newly learned information is maintained over time, and whether this effect could translate into real increases in academic achievement.

## Author Contributions

SS, TB, and LR developed the concept of the study and all authors were involved in study design. SS, EB, and TB collected and processed the data. All authors planned and performed data analyses. All authors wrote components of the manuscript and approved the final version.

## Conflict of Interest Statement

The authors declare that the research was conducted in the absence of any commercial or financial relationships that could be construed as a potential conflict of interest.

The reviewer FP and the handling Editor declared their shared affiliation, and the handling Editor states that the process nevertheless met the standards of a fair and objective review.

## References

[B1] Adan A. Prat G. Fabbri M. Sànchez-Turet M. (2008). Early effects of caffeinated and decaffeinated coffee on subjective state and gender differences. *Prog. Neuropsychopharmacol. Biol. Psychiatry* 32 1698–1703. 10.1016/j.pnpbp.2008.07.005 18675877

[B2] Adan A. Serra-Grabulosa J. M. (2010). Effects of caffeine and glucose, alone and combined, on cognitive performance. *Hum. Psychopharmacol.* 25 310–317. 10.1002/hup.1115 20521321

[B3] Anderson M. J. Petros T. V. Beckwith B. E. Mitchell W. W. Fritz S. (1991). Individual differences in the effect of time of day on long-term memory access. *Am. J. Psychol.* 104 241–255. 10.2307/1423157

[B4] Bailey S. L. Heitkemper M. M. (2001). Circadian rhythmicity of cortisol and body temperature: morningness-eveningness effects. *Chronobiol. Int.* 18 249–261. 10.1081/CBI-100103189 11379665

[B5] Borota D. Murray E. Keceli G. Chang A. Watabe J. M. Ly M. (2014). *Post-Study Caffeine Administration Enhances Memory Consolidation in Humans.* London: Nature Publishing Group 10.1038/nn.3623 PMC5909971 24413697

[B6] Brice C. Smith A. (2001). The effects of caffeine on simulated driving, subjective alertness and sustained attention. *Hum. Psychopharmacol.* 16 523–531. 10.1002/hup.327 12404548

[B7] Bugg J. M. DeLosh E. L. Clegg B. A. (2006). Physical activity moderates time-of-day differences in older adults’. Working memory performance. *Exp. Aging Res.* 32 431–446. 10.1080/03610730600875833 16982572

[B8] Capek S. Guenther R. K. (2009). Caffeine’s effects on true and false memory. *Psychol. Rep.* 104 787–795. 10.2466/PR0.104.3.787-795 19708406

[B9] Chelminski I. Ferraro F. R. Petros T. Plaud J. J. (1997). Horne and Ostberg questionnaire: a score distribution in a large sample of young adults. *Pers. Individ. Dif.* 23 647–652. 10.1016/S0191-8869(97)00073-1

[B10] Cooper C. J. (1973). Anatomical and physiological mechanisms of arousal with specific reference to the effects of exercise. *Ergonomics* 16 601–609. 10.1080/00140137308924551 4772986

[B11] Delpouve J. Schmitz R. Peigneux P. (2014). ScienceDirect. *Cortex* 58 18–22. 10.1016/j.cortex.2014.05.006 24954854

[B12] Dietrich A. Audiffren M. (2011). The reticular-activating hypofrontality (RAH) model of acute exercise. *Neurosci. Biobehav. Rev.* 35 1305–1325. 10.1016/j.neubiorev.2011.02.001 21315758

[B13] Einöther S. J. L. Giesbrecht T. (2012). Caffeine as an attention enhancer: reviewing existing assumptions. *Psychopharmacology* 225 251–274. 10.1007/s00213-012-2917-4 23241646

[B14] Hasher L. Goldstein D. May C. P. (2005). It’s about time: circadian rhythms, memory and aging”, in *Human Learning and Memory: Advances in Theory and Application: The 4th Tsukuba International Conference on Memory*. eds Izawa C. Ohta N. (Mahwah, NJ: Erlbaum Associates Publishers) 199–217.

[B15] Heckman M. A. Weil J. de Mejia E. G. (2010). Caffeine (1, 3, 7-trimethylxanthine) in foods: a comprehensive review on consumption, functionality, safety, and regulatory matters. *J. Food Sci.* 75 R77–R87. 10.1111/j.1750-3841.2010.01561.x 20492310

[B16] Hidalgo M. P. L. Zanette C. B. Pedrotti M. Souza C. M. Nunes P. V. Fagundes C. M. L. (2004). Performance of chronotypes on memory tests during the morning and the evening shifts. *Psychol. Rep.* 95 75–85. 10.2466/PR0.95.5.75-85 15460360

[B17] Horne J. A. Ostberg O. (1976). A self-assessment questionnaire to determine morningness-eveningness in human circadian rhythms. *Int. J. Chronobiol.* 4 97. 1027738

[B18] Hourihan K. L. Benjamin A. S. (2013). State-based metacognition: how time of day affects the accuracy of metamemory. *Memory* 22 553–558. 10.1080/09658211.2013.804091 23742008 PMC3818346

[B19] Hung T.-M. Tsai C.-L. Chen F.-T. Wang C.-C. Chang Y.-K. (2013). The immediate and sustained effects of acute exercise on planning aspect of executive function. *Psychol. Sport Exerc.* 14 728–736. 10.1016/j.psychsport.2013.05.004

[B20] Intons-Peterson M. J. Rocchi P. West T. McLellan K. Hackney A. (1998). Aging, optimal testing times, and negative priming. *J. Exp. Psychol.* 24 362.

[B21] Knight M. Mather M. (2013). Look out – it’s your off-peak time of day! time of day matters more for alerting than for orienting or executive attention. *Exp. Aging Res.* 39 305–321. 10.1080/0361073X.2013.779197 23607399 PMC4067093

[B22] May C. P. Hasher L. Foong N. (2005). Implicit Memory. Age, and Time of Day. *Psychol. Sci.* 16 96–100. 10.1111/j.0956-7976.2005.00788.x 15686574 PMC1751473

[B23] May C. P. Hasher L. Stoltzfus E. R. (1993). Optimal time of day and the magnitude of age differences in memory. *Psychol. Sci.* 4 326–330. 10.1111/j.1467-9280.1993.tb00573.x

[B24] McGaugh J. L. (2000). Memory–a century of consolidation. *Science* 287 248–251. 10.1126/science.287.5451.248 10634773

[B25] McMorris T. Sproule J. Turner A. Hale B. (2011). Acute, intermediate intensity exercise, and speed and accuracy in working memory tasks: a meta-analytical comparison of effects. *Physiol. Behav.* 102 421–428. 10.1016/j.physbeh.2010.12.007 21163278

[B26] Michael N. Johns M. Owen C. Patterson J. (2008). Effects of caffeine on alertness as measured by infrared reflectance oculography. *Psychopharmacology* 200 255–260. 10.1007/s00213-008-1202-z 18537025

[B27] Nehlig A. (2010). Is caffeine a cognitive enhancer? *J. Alzheimer’s Dis.* 20 85–94. 10.3233/JAD-2010-091315 20182035

[B28] Nehlig A. Boyet S. (2000). Dose–response study of caffeine effects on cerebral functional activity with a specific focus on dependence. *Brain Res.* 858 71–77. 10.1016/S0006-8993(99)02480-4 10700599

[B29] Petros T. V. Beckwith B. E. Anderson M. (1990). Individual differences in the effects of time of day and passage difficulty on prose memory in adults. *Br. J. Psychol.* 81 63–72. 10.1111/j.2044-8295.1990.tb02346.x

[B30] Randler C. Schaal S. (2010). Morningnessâ€“eveningness, habitual sleep-wake variables and cortisol level. *Biol. Psychol.* 85 14–18. 10.1016/j.biopsycho.2010.04.006 20450953

[B31] Reber P. J. (2013). The neural basis of implicit learning and memory: a review of neuropsychological and neuroimaging research. *Neuropsychologia* 51 2026–2042. 10.1016/j.neuropsychologia.2013.06.019 23806840

[B32] Rowe G. Valderrama S. Hasher L. Lenartowicz A. (2006). Attentional disregulation: a benefit for implicit memory. *Psychol. Aging* 21 826–830. 10.1037/0882-7974.21.4.826 17201503 PMC1858627

[B33] Ryan L. Hatfield C. Hofstetter M. (2002). Caffeine reduces time-of-day effects on memory performance in older adults. *Psychol. Sci.* 13 68–71. 10.1111/1467-9280.00412 11892781

[B34] Ryan L. Ostergaard A. Norton L. Johnson J. (2001). Search and selection processes in implicit and explicit word-stem completion performance in young, middle-aged, and older adults. *Mem. Cogn.* 29 678–690. 10.3758/BF03200470 11531223

[B35] Segal S. K. Stark S. M. Kattan D. Stark C. E. Yassa M. A. (2012). Norepinephrine-mediated emotional arousal facilitates subsequent pattern separation. *Neurobiol. Learn. Mem.* 97 465–469. 10.1016/j.nlm.2012.03.010 22498686 PMC3517207

[B36] Simons S. B. Caruana D. A. Zhao M. Dudek S. M. (2012). Caffeine-induced synaptic potentiation in hippocampal CA2 neurons. *Nat. Neurosci.* 15 23–25. 10.1038/nn.2962 PMC3245784 22101644

[B37] Smith A. (2005). Effects of repeated doses of caffeine on mood and performance of alert and fatigued volunteers. *J. Psychopharmacol.* 19 620–626. 10.1177/0269881105056534 16272184

[B38] Walters E. R. Lesk V. E. (2015). Time of day and caffeine influence some neuropsychological tests in the elderly. *Psychol. Assess.* 27 161 10.1037/a0038213 25346994

